# Gamma Tocotrienol Protects Mice From Targeted Thoracic Radiation Injury

**DOI:** 10.3389/fphar.2020.587970

**Published:** 2020-11-12

**Authors:** Vidya P. Kumar, Sasha Stone, Shukla Biswas, Neel Sharma, Sanchita P. Ghosh

**Affiliations:** Armed Forces Radiobiology Research Institute, Uniformed Services University of the Health Sciences, Bethesda, MD, United States

**Keywords:** gamma tocotrienol, partial body irradiation, small animal radiation research platform, radiation injury to normal lungs, prophylactic treatment, Thoracic irradiation

## Abstract

Radiation injury will result in multiorgan dysfuntion leading to multiorgan failure. In addition to many factors such as radiation dose, dose rate, the severity of the injury will also depend on organ systems which are exposed. Here, we report the protective property of gamma tocotrienol (GT3) in total as well as partial body irradiation (PBI) model in C3H/HeN male mice. We have carried out PBI by targeting thoracic region (lung-PBI) using Small Animal Radiation Research Platform, an X-ray irradiator with capabilities of an image guided irradiation with a variable collimator with minimized exposure to non-targeted tissues and organs. Precise and accurate irradiation of lungs was carried out at either 14 or 16 Gy at an approximate dose rate of 2.6 Gy/min. Though a low throughput model, it is amenable to change the field size on the spot. No damage to other non-targeted organs was observed in histopathological evaluation. There was no significant change in peripheral blood counts of irradiated mice in comparison to naïve mice. Femoral bone marrow cells had no damage in irradiated mice. As expected, damage to the targeted tissue was observed in the histopathological evaluation and non-targeted tissue was found normal. Regeneration and increase of cellularity and megakaryocytes on GT3 treatment was compared to significant loss of cellularity in saline group. Peak alveolitis was observed on day 14 post-PBI and protection from alveolitis by GT3 was noted. In irradiated lung tissue, thirty proteins were found to be differentially expressed but modulated by GT3 to reverse the effects of irradiation. We propose that possible mode of action of GT3 could be Angiopoietin 2-Tie2 pathway leading to AKT/ERK pathways resulting in disruption in cell survival/angiogenesis.

## Introduction

Gamma tocotrienol (GT3), a naturally occurring isoform of vitamin E (Ghosh et al., 2008; Singh and Hauer-Jensen, 2016), a fat soluble antioxidant has been shown to have radioprotective properties (Ghosh et al., 2009). Further studies showed recovery of hematopoietic stem cells (HSC) and progenitor cells (HPC) in GT3 treated irradiated mice suggesting the prophylactic efficacy of GT3 was through protection of hematopoietic tissue and prevention of persistent DNA damage (Ghosh et al., 2009; Kulkarni et al., 2010). In addition to HSC and HPCs, it has been shown that GT3 mobilizes endothelial progenitor cells (EPCs) as well, by significantly increasing the levels of G-CSF and VEGF, thus ameliorating the damage to the hematopoietic system (Ray et al., 2013). The protective efficacy of GT3 mediated through G-CSF was demonstrated by abrogating the survival of mice by neutralization of G-CSF using the antibodies (Kulkarni et al., 2013).

In addition to protection of hematopoietic injury, GT3 has been shown to ameliorate gastrointestinal (GI) injury reducing the vascular oxidative stress after TBI (Berbee et al., 2009). In the case of GI injury, plasma citrulline levels were shown to increase in GT3 group on day 7 post-TBI, indicating recovery of intestinal mucosa. This result was confirmed by reduced load of bacterial DNA in the liver in the GT3 pre-treated group when compared to vehicle group. The molecular evidence of the protection of the intestinal crypts has been shown to be due to upregulation of anti-apoptotic and downregulation of pro-apoptotic factors (Suman et al., 2013). Radiation injury to the microvasculature reduces availability of the eNOS cofactor tetrahydrobiopterin (BH4) during early post-radiation phase which in turn results in uncoupling of eNOS increasing the oxidative stress (Landmesser et al., 2003; Cosentino et al., 2008). Pre-treatment with GT3 seems to have protected the tissue by regulating the key proteins in the BH4 pathway (Berbee et al., 2011). Expression levels of several cell signaling proteins in Wnt pathway was attributed to GT3’s radioprotective efficacy in mice (Cheema et al., 2018). GT3 seems to have pleiotropic effect that restores or modulates expression of key proteins that regulate various prime processes in the cell such as DNA replication, recombination and repair, development of B-cells and various immunological responses to radiation insult (Cheema et al., 2018).

Previously published studies with GT3 discussed so far were performed using TBI, where mice were exposed to whole body irradiation and lethality was primarily due to either hematopoietic failure or a combination of hematopoietic and gastrointestinal injury. Radiation lethality may occur due to multi-organ failure, depending on total absorbed dose following a radiological event. Historically, there has been a continued interest in studying the efficacy of radiation countermeasures using partial body irradiation (PBI) in order to understand the efficacy from organ specific injuries. Characterization of death due to certain organ specific injury was carried out by exposing animals to PBI such as head, trunk or lower body (pelvis, legs and tail) and dose dependent mortality was studied by probit analysis and compared it to TBI (Sato et al., 1972). The authors concluded that PBI didn’t have much influence on the pattern of “daily death” or the period of peak mortality, thus characterizing the death based on syndromes as oral death for head exposure, intestinal death for trunk exposure and bone marrow death for the lower body exposure (Sato et al., 1972). Lung injury often involves degeneration and regeneration of epithelial cells, activation and infiltration of inflammatory cells, disruption of microvasculature, and excessive matrix protein production leading to thickening of the epithelial walls (McDonald et al., 1995; Fan et al., 2001; Mehta, 2005). Radiation-induced lung injury is typically manifested as pneumonitis or fibrosis within months to few years after exposure (McDonald et al., 1995; Fan et al., 2001; Mehta, 2005), however, radiation injury to the thorax may occur in early time points after exposure. Mostly, all previously published studies were performed using CD2F1 mouse model to determine the prophylactic efficacy of GT3 (Ghosh et al., 2009; Kulkarni et al., 2010; Ray et al., 2013; Singh et al., 2016). In this study, we used small animal radiation research platform (SARRP) to evaluate radiation-induced lung injury during 1-30 days post-irradiation in C3H/HeN mice and determined the prophylactic efficacy of GT3 in ameliorating lung injury through pathological and biochemical analysis. We also validated prophylactic efficacy of GT3 against the whole body gamma-radiation. In addition, we have evaluated the role of Ang-2-Tie-2-AKT pathway in the protection of lungs by pre-treatment with GT3.

## Materials and Methods

### Mice

Twelve to fourteen week old C3H/HeN male mice used in these studies were purchased from Envigo Corporation, Indianapolis, IN, USA. The mice were housed in the Armed Forces Radiobiology Research Institute’s (AFRRI) vivarium accredited by the Association for Assessment and Accreditation of Laboratory Animal Care-International. The animals received Harlan Teklad Rodent Diet 8604 and acidified water (pH 2.5 – 3.0) ad libitum and were housed under 12 h light/dark cycle and were acclimatized for 2 weeks before the start of each study. All procedures in these studies were performed under an approved protocol by the Department of Defense Institutional Animal Care Use Committee (IACUC) (Ghosh et al., 2009).

### GT3 Formulation

Pure GT3 was obtained from Yasoo Health Inc. (Johnson City, TN, United States) (Ghosh et al., 2009) and American River Nutrition (Hadley, MA, United States). GT3 and Tween80^®^ (final concentration 5%) were dissolved separately in small volume of ethanol (to enable uniform mixing) and mixed together and then spin-dried under vacuum. Required volume of saline was added to the tube to achieve a final concentration of either 100 or 200 mg GT3 in 0.1 ml.

### Total Body Irradiation (TBI)

In the total body irradiation studies, the experimental animals received a single exposure of Co-60 gamma at an estimated dose rate of 0.6 Gy/min in the AFRRI radiation facility. The mice were placed in ventilated Lucite^™^ boxes arranged in an array using plastic racks during the exposure. The alanine electron spin resonance (ESR) dosimetry system (ASTM Standard E 1607‐94, 1994) was used to measure dose rates to water in cores of acrylic mouse phantoms as described earlier (Ghosh et al., 2009). The radiation field was uniform within ±2%.

### Thoracic Radiation (Lung-Partial Body Irradiation)

The experimental animals received an anterior-posterior-posterior-anterior (AP-PA) exposure of X-ray at an estimated dose rate of 2.6 Gy/min in the AFRRI’s SARRP radiation facility (Cho & Kazanzides, 2012). Ionization chambers and alanine dosimeters with traceability to national standards, and mouse phantoms were used to measure radiation doses at 220 kVp and 13 mA. Radiation doses were checked daily using ionization chambers. Determinations of coordinates and isocenter to establish the field of exposure was done using CT scans of the mice. Mice were anesthetized during irradiation using isoflurane. A fluoroscopic X-ray image using portal imaging camera was used to confirm the desired isocenter and field of exposure (Supplementary Figure S1). For lung-PBI, C3H/HeN male mice were exposed to 14 or 16 Gy doses of radiation to the lung area (below the neck to diaphragm). Irradiated mice were administered either saline or GT3 (200 mg/kg, SC) 24 h prior to PBI. All the animals were transferred to their respective cages on recovery from anesthesia with free access to food and acidified water, and monitored daily for 30 days (up to three times a day when necessary) for body weight loss, ruffled fur or any behavioral abnormalities. Mice showing signs of moribundity (significant weight loss, ruffled fur, difficulty in breathing and moving) were euthanized immediately by CO_2_ inhalation exposure and cervical dislocation according to the AFRRI IACUC protocol guidelines.

### Survival Study in Total Body Irradiation Model Using Cobalt-60 Gamma Radiation

C3H/HeN male mice were weighed and randomly distributed into various groups (n = 16/group). There were 16 mice per radiation dose (7.5, 8, 8.5 or 9 Gy at an approximate rate of 0.6 Gy/min) and treatment group (saline, GT3 at either 100 or 200 mg/kg body weight). The mice received SC administration (under the nape of the neck) of either GT3 or saline (the vehicle) 24 h prior to TBI. After irradiation, mice were returned to their original cages with free access to food and acidified water, and monitored daily for 30 days (up to three times a day when necessary) for body weight loss and clinical symptoms of distress and pain. Mice showing signs of moribundity were euthanized immediately by CO_2_ inhalation exposure and cervical dislocation according to the AFRRI IACUC protocol guidelines. Surviving animals were euthanized at the completion of the observational period.

### Blood and Tissue Collection

Blood and tissues were collected from the experimental animals at various time points post-PBI for analyses. GT3 (200 mg/kg) or saline (n = 12 per group) were administered SC 24 h prior to irradiation. In addition to the irradiated groups of animals, blood and tissues from an age-matched naïve group (n = 12) was collected at each time point. Blood was collected from inferior vena cava under anesthesia on days 1, 14, and 30. Complete blood counts (CBC) and differential analysis was performed using HESKA Element HT (TM) 5 Analyzer system. Sternum, heart, lungs, jejunum and kidney were collected and either processed for histopathological findings or snap frozen in liquid nitrogen. Femurs were collected for isolation of bone marrow cells to carry out Colony Forming Unit (CFU) Assay as described below.

### Femoral BM Colony Forming Unit Assay

Clonogenicity of mouse bone marrow cells was quantified in standard semisolid cultures using 1 ml of Methocult GF+ system for mouse cells (Stem Cell Technologies Inc., Vancouver, BC) as reported earlier (Kumar et al., 2018). Briefly, colony forming units (CFU) were assayed on days 1, 14, and 30 from irradiated groups and the age-matched naïve mice. Cells from three femurs from different animals were pooled, washed twice with IMDM and seeded at 1 to 5 × 10^4^ cells per dish in 35-mm^2^ cell culture dishes (BD Biosciences). Each sample was plated in duplicate to be scored 14 days after plating. Colonies which included Granulocyte-macrophage colony forming units (CFU-GM), granulocyte-erythrocyte-monocyte-macrophage CFU (CFU-GEMM), colony-forming unit-erythroid (CFU-E) and erythroid burst-forming units (BFU-E) were counted 14 days after plating using a Nikon TS100F microscope. Fifty or more cells were considered one colony. Data are expressed as mean ± standard error of mean (SEM). Statistical significance was determined between irradiated vehicle treated and GT3-treated groups.

### Sternal Histopathology

Following blood collection, animals were euthanized, and the sterna were collected. The sterna were fixed in a 20:1 volume of fixative (10% buffered formalin) to tissue for at least 24 h and up to 7 days. Fixed sterna were decalcified for 3 h in 12–18% sodium EDTA (pH 7.4–7.5) and specimens dehydrated using graded ethanol concentrations and embedded in paraffin. Longitudinal 5 μm sections were stained with regular hematoxylin and eosin (H&E) stain. The bone marrow was evaluated *in situ* within sternebrae and graded for megakaryocyte numbers averaged per 10 high power fields at 40× magnification using a BX41 Olympus microscope (Minneapolis, MN) (Kumar et al., 2018).

### Histopathology of Lung, Heart and Jejunal Sections

Post-blood collection and euthanasia, collected tissue were fixed in 10% buffered formalin. Tissue sections of heart (longitudinal), lung (standard orientation, embedded on largest area) and jejunum (circular 5 μm sections) were stained with regular hematoxylin and eosin (H&E) stain. A board-certified veterinary pathologist conducted blinded histopathological evaluation of these samples (Sharma et al., 2020).

### Proteome Profiler Array

Frozen lung tissue samples were homogenized using the Bullet Blender Tissue Homogenizer (Next Advance, Inc, Troy, NY, United States) in RIPA buffer with protease inhibitors, and total protein content of supernatants was determined by bicinchoninic acid assay. Proteome Profiler Mouse XL Cytokine Arrays (R&D Systems, Minneapolis, MN) were performed per the manufacturer’s instructions. For each array, supernatants from three individual animals were spotted in duplicate, and arrays were performed per condition. Naïve group was used as a control. High resolution images of the blots were analyzed using Western Vision’s HLImage++ software (designed to analyze arrays from R&D systems) and GraphPad Prism 7 software (GraphPad Software, 7825 Fay Av., Suite 230, La Jolla, CA 92037, United States) was used to plot the data.

### Phosphorylated Proteins From AKT Signaling Pathway

PathScan^®^ AKT Signaling Antibody Array Kit #9700 (Cell Signaling Technology, Inc., Danvers, MA, United States) was used to detect the differential levels of phosphorylated proteins in lung lysates. Frozen lung tissue from naïve and the two irradiated groups (saline and GT3 treated) were homogenized as mentioned earlier for proteome array. The PathScan^®^ kit was used as per the manufacturer’s instructions. The data was collected and analyzed using the Odyssey CLx instrument and LiCOR analysis software (LiCor Biosciences).

### Immunohistochemistry

Immunohistochemistry involved two preparatory steps – antigen retrieval and staining (Sharma et al., 2020). Unstained sections (4 μM) of formalin-fixed, paraffin-embedded specimens were deparaffinized in xylene, and hydrated in a series of ethanol dilutions (100, 95, and 80% ethanol in water) with final rinse in distilled water. Slides were then incubated for about 40 min in citrate buffer (10 mM Citric Acid, 0.05% Tween 20, pH 6.0) at 95–100°C in a water bath/steamer. At the end of this incubation, the slides were allowed to cool to room temperature, followed by rinsing with PBS-Tween 20 (0.1%). The sections were then processed for immunostaining beginning with blocking (1% BSA, 0.5% TX100 in PBS) for 30 min followed by incubation with primary antibody (Ang-2 (Cell Signaling Technology 2948S), pAKT (Cell Signaling Technology 9271S), pP38 (Cell Signaling Technology 9211S), Tie-2 (Cell Signaling Technology 09D10), pTie-2 (R&D Systems AF3909) at 4°C overnight. After washing the slides with wash buffer (1× PBS, 0.1% triton) five times for 5 min each, the sections were incubated with the secondary antibody for 2 h at room temperature. After secondary antibody incubation again slides were washed five times for 5 min each. Mounting of sections were carried out using Abcam kit (BrightMount- Aqueous Mounting Medium for Fluorescent Staining ab103746). The sections were imaged on Zeiss 700 confocal microscope.

### Statistical Analysis

Survival data was plotted as a Kaplan-Meier plot and statistical significance of the survival differences was determined by log-rank test using GraphPad Prism 7 software (GraphPad Software, 7825 Fay Av., Suite 230, La Jolla, CA 92037, United States). For comparison of two different groups, statistical significance was determined using the Holm-Sidak method, with alpha = 0.05. Each pair was analyzed individually, without assuming a consistent standard deviation (SD).

## Results

### Pre-Treatment of GT3 has Significant Survival Benefit in C3H/HeN Mice Exposed to Total Body Gamma Radiation

**FIGURE 1:**
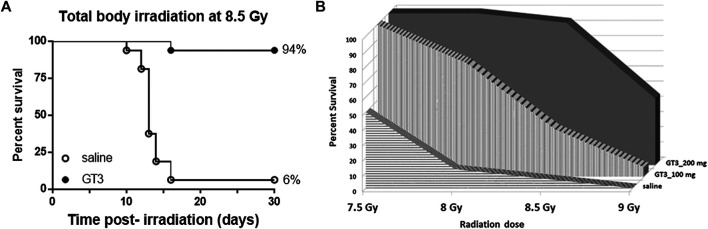
Survival of C3H/HeN male mice following total-body irradiation at an estimated rate of 0.6 Gy/min and SC administration of a single dose of either GT3 or saline as vehicle at 24 h prior to TBI. **(A)** Kaplan-Meier survival curves were plotted using GraphPad software; n = 16 mice per group and trend in survival at 8.5 Gy is compared between vehicle (○) and GT3 (200 mg/kg) group (●) (log-rank test *p* < 0.0001). **(B)** Dose response in terms of radiation doses (7.5, 8, 8.5, and 9 Gy) and doses of GT3 (100 and 200 mg/kg) were evaluated in a 30-days survival study comparing to saline treated group (n = 16/dose of radiation/treatment group).

To evaluate efficacy of GT3 as a prophylactic countermeasure in C3H/HeN mice, 16 mice/group were administered either saline or GT3 (200 mg/kg) 24 h prior to whole body gamma irradiation. The survival efficacy was evaluated at an 8.5 Gy dose of radiation. When compared with saline group, GT3 group had 94% survival whereas only 6% in saline group, resulting in the Kaplan-Meier curve comparison by Log-rank test p value to be ≤0.0001 (Figure 1A). Four doses of radiation were tested to establish dose response on survival. Two doses of GT3 (100 and 200 mg/kg) were tested along with saline group for this study (n = 16 mice/group). As shown in the Figure 1B, at the lowest dose of radiation (7.5 Gy) tested, saline group had 50% survival whereas GT3 groups had 100% survival. Interestingly, percent survival in the drug treated group was higher at 200 mg/kg dose compared to the lower dose (100 mg/kg) at all radiation doses (8, 8.5, and 9 Gy). At 8 Gy 75% and 100% survival was observed at 100 and 200 mg/kg, respectively. At 8.5 Gy the difference was wider with 31.25% survival with 100 mg/kg but the higher GT3 dose group had much higher survival (94%). 9 Gy radiation dose was a supra-lethal to the saline group as all animals died before day 30, GT3 at 100 mg/kg also had significant mortality (survival of only 1/16 mice) in comparison to GT3 at 200 mg/kg group with 44% survival.

### No Effect on Peripheral Blood Cell Counts After Targeted Irradiation in Lung

Blood was collected from naïve and 14 and 16 Gy lung-irradiated mice at different time points and was analyzed for CBC. There was no significant difference in the cell counts between the naïve and two irradiated groups (saline and GT3 treated) for both radiation doses (Supplementary Figure S2).

### No Effect on Bone Marrow Progenitor Cell Counts Post-Partial Body Irradiation in Lung

Femoral bone marrow from naïve and 14 and 16 Gy lung-PBI groups were collected on days 1, 14, and 30 post-PBI. Clonogenic assays were carried out to evaluate the extent of damage caused by irradiation, if any. CFU assays measured CFU-GM and CFU-GEMM and BFU-E to evaluate the function of hematopoietic cells. Based on the CFU counts, there was no significant effect of irradiation on the femoral bone marrow (Supplementary Figure S3) as this part of the body was spared from irradiation.

### Significant Recovery of Sternal Bone Marrow Cellularity Post-Partial Body Irradiation in Lung

Bone marrow architecture and cellularity of mice were evaluated on sternum samples collected on days 1, 14, and 30 post-lung PBI. The extent of recovery from radiation damage was estimated from the H&E stained slides by quantifying the megakaryocytes. Megakaryocytes were evaluated by averaging the number of cells per 10 (40×) high-powered fields (HPFs).

**FIGURE 2:**
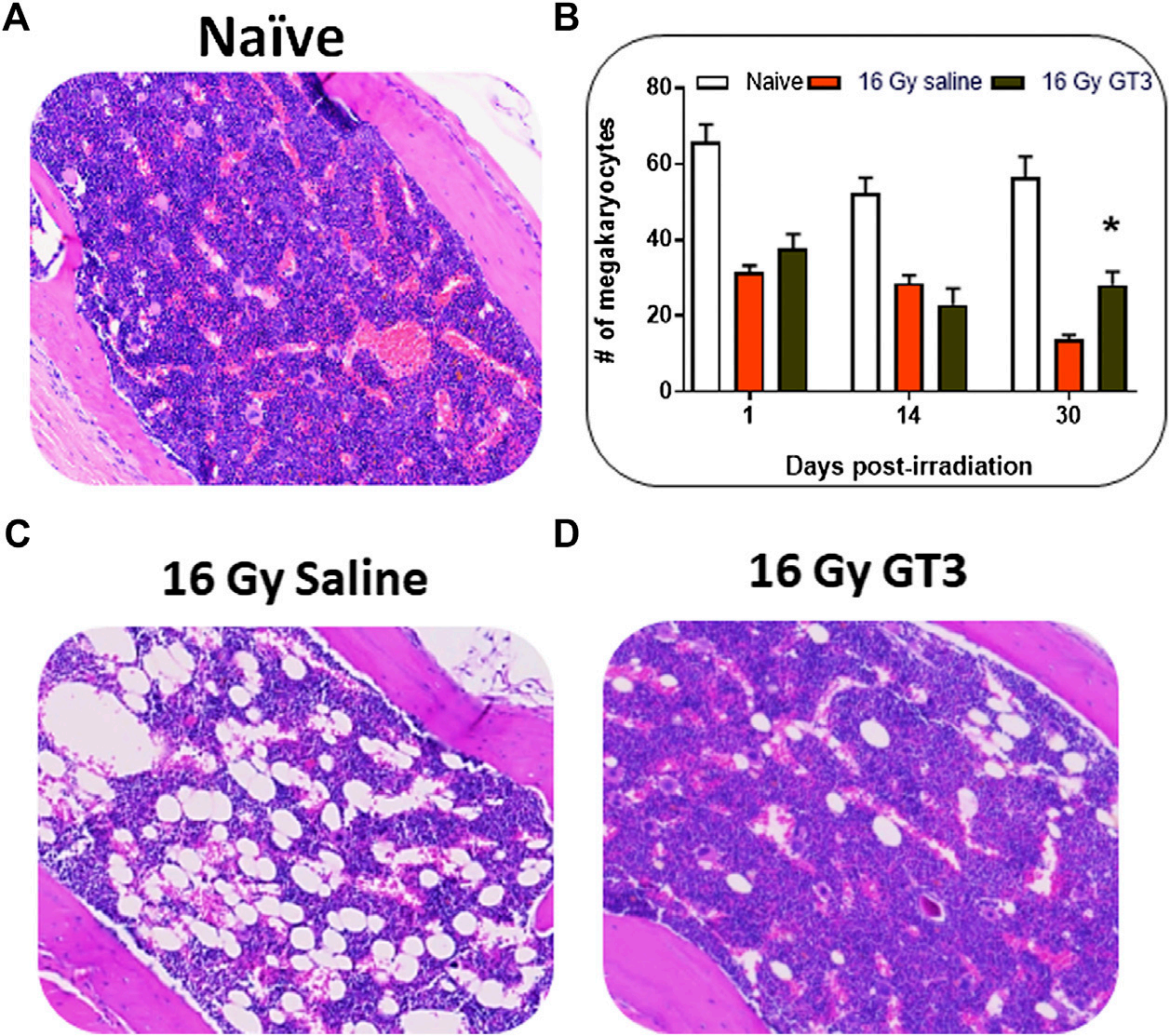
GT3 treatment promoted sternal bone marrow hematopoietic cell recovery after 16 Gy PBI (to thorax) when administered 24 h prior to PBI. Representative sternal bone marrow sections are shown for naïve **(A)**, and from saline **(C)** and GT3 **(D)** treated 16 Gy irradiated mice from day 30 post-PBI. Bone marrow megakaryocyte numbers were quantitated from histological sections from days 1, 14, and 30 post-PBI **(B)**. Significant increase in bone marrow cellularity and megakaryocytes **(B)** were observed on day 30 post-PBI in the GT3 treatment group. Data represented are mean ± standard error of the mean (SEM) for n = 12 mice.

When compared to naive controls, irradiated samples showed significant damage. On day 30 post-PBI, the % cellularity in the GT3 treated PBI group was significantly higher than that of the saline treated group, which was reflected in the number of megakaryocytes as well (Figure 2).

### Histopathology of Jejunum

Jejunal tissue samples from naïve and lung-PBI (14 and 16 Gy) groups (saline and GT3 treated) were collected on days 1, 14, and 30 post-PBI and subjected to histopathological evaluation of H&E stained cross sections. There was no difference in the viable crypt count observed between naïve and saline or GT3 treated irradiated (14 and 16 Gy) groups (Supplementary Figure S4A).

### Histopathology of Heart

Heart tissue from naïve and irradiated (14 and 16 Gy lung-PBI) were evaluated by the board certified pathologist for abnormalities and radiation damage. H&E stained sections were evaluated for epicardial thickening and fibrosis, myocardial fibrosis, and coronary artery disease. No effects attributable to irradiation were observed in the mice that received either a 14 or 16 Gy dose when observed at days 1, 14, and 30 post-irradiation (Supplementary Figure S4B).

### Histopathological Analysis of Lung Sections

**FIGURE 3:**
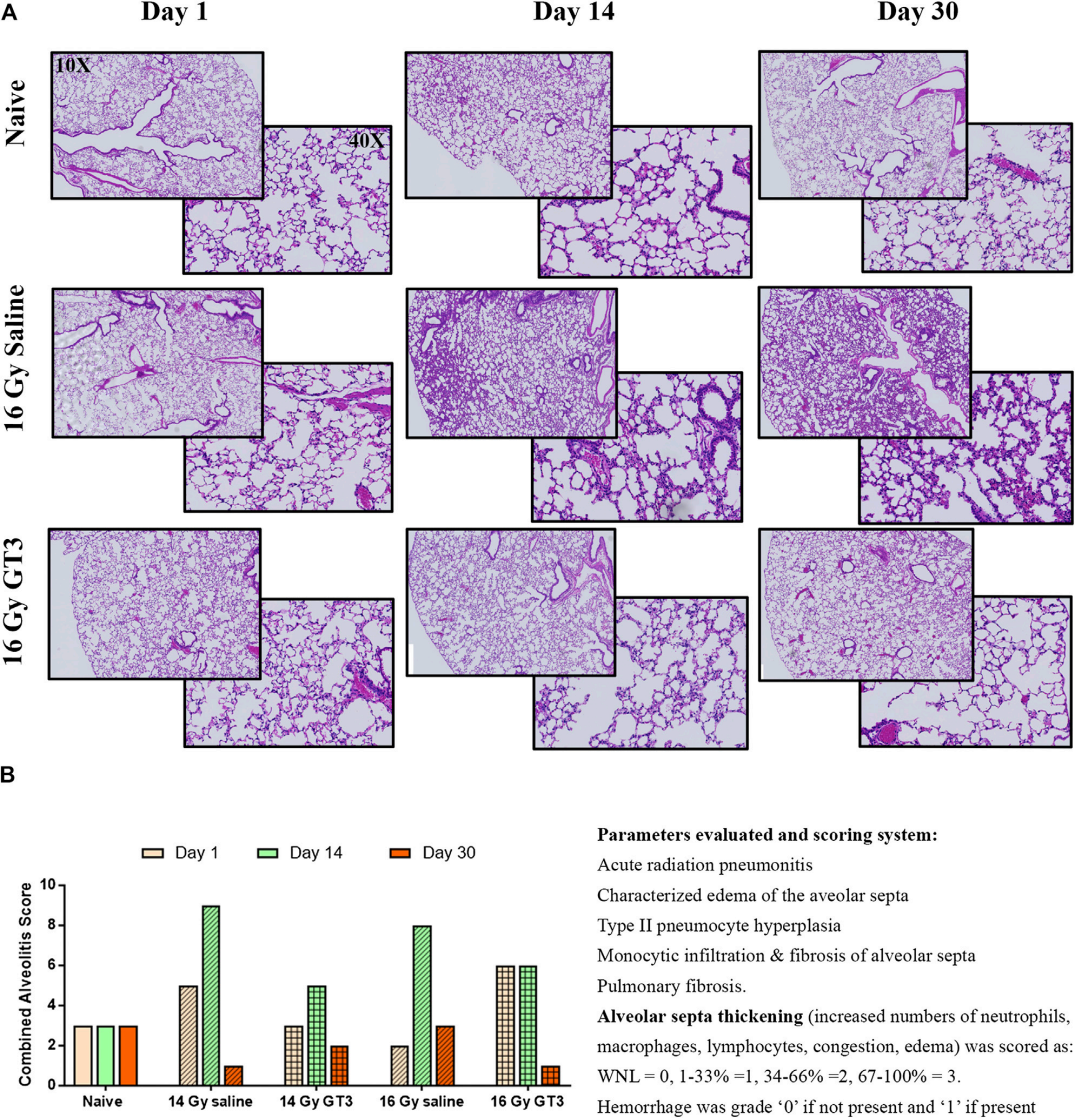
Histopathological evaluation of lungs irradiated at 14 and 16 Gy. Representative H&E stained lung sections from 16 Gy irradiation groups in (shown as 10 and 40 magnification) **(A)** were evaluated for signs of irradiation effects such as characteristic edema of the aveolar septa, type II pneumocyte hyperplasia, monocytic infiltration and fibrosis of alveolar septa, and hyaline membranes that line alveoli; and pulmonary fibrosis. **(B)** Thickening of alveolar septa (increased numbers of neutrophils, macrophages, lymphocytes, congestion, and edema) was scored as: WNL = 0, 1–33% = 1, 34–66% = 2, 67–100% = 3. Hemorrhage was grade “0” if not present and “1” if present regardless of amount.Severe damage to the lung tissue on irradiation was observed on all three days and in both 16 and 14 Gy irradiation doses in saline treated groups. Lung tissue from GT3 treated groups showed less damage and recovery by day 30.

H&E stained sections of lung tissue from naïve and irradiated groups (lung-PBI at 14 and 16 Gy) were evaluated by the Board Certified Veterinary Pathologist. The parameters evaluated were for acute radiation pneumonitis, characterized edema of the aveolar septa, Type II pneumocyte hyperplasia, monocytic infiltration and presence of fibrosis (Figure 3A). Alveolitis was quantified as a cumulative score of thickening of alveolar septa and extent of hemorrhage (Figure 3B). Thickening of alveolar septa as a result of increased numbers of neutrophils, macrophages, lymphocytes causing congestion and edema was scored as: WNL = 0, 1–33% = 1, 34–66% = 2, 67–100% = 3. Hemorrhage was graded as 0 if not present and 1 if present regardless of the amount. Alveolar septa was observed to be multi-focally expanded by neutrophils, macrophage, lymphocytes, congestion, and rarely edema. The peak of this inflammation was seen at day 14 post-PBI. Protection from alveolitis by GT3 was observed on days 14 post-PBI when irradiated at both 14 and 16 Gy. However, by day 30, all irradiated groups showed recovery. In many lung sections on day 14, alveolar septa were multifocally expanded by neutrophils, macrophage, lymphocytes, congestion, and rarely edema. At 30 days post-irradiation, there was much less inflammation, but of interest, large, foamy macrophages were present in the lungs of irradiated mice which were not as readily apparent in the naïve controls. However, on day 1 post-PBI being too short a time to see structural damage, variations in 14 Gy saline having higher score than 16 Gy saline can be attributed to the animal to animal variation in response to the inflammatory stimuli.

### Differential Expression of Inflammatory Cytokines/Chemokines due to Radiation Injury

**FIGURE 4:**
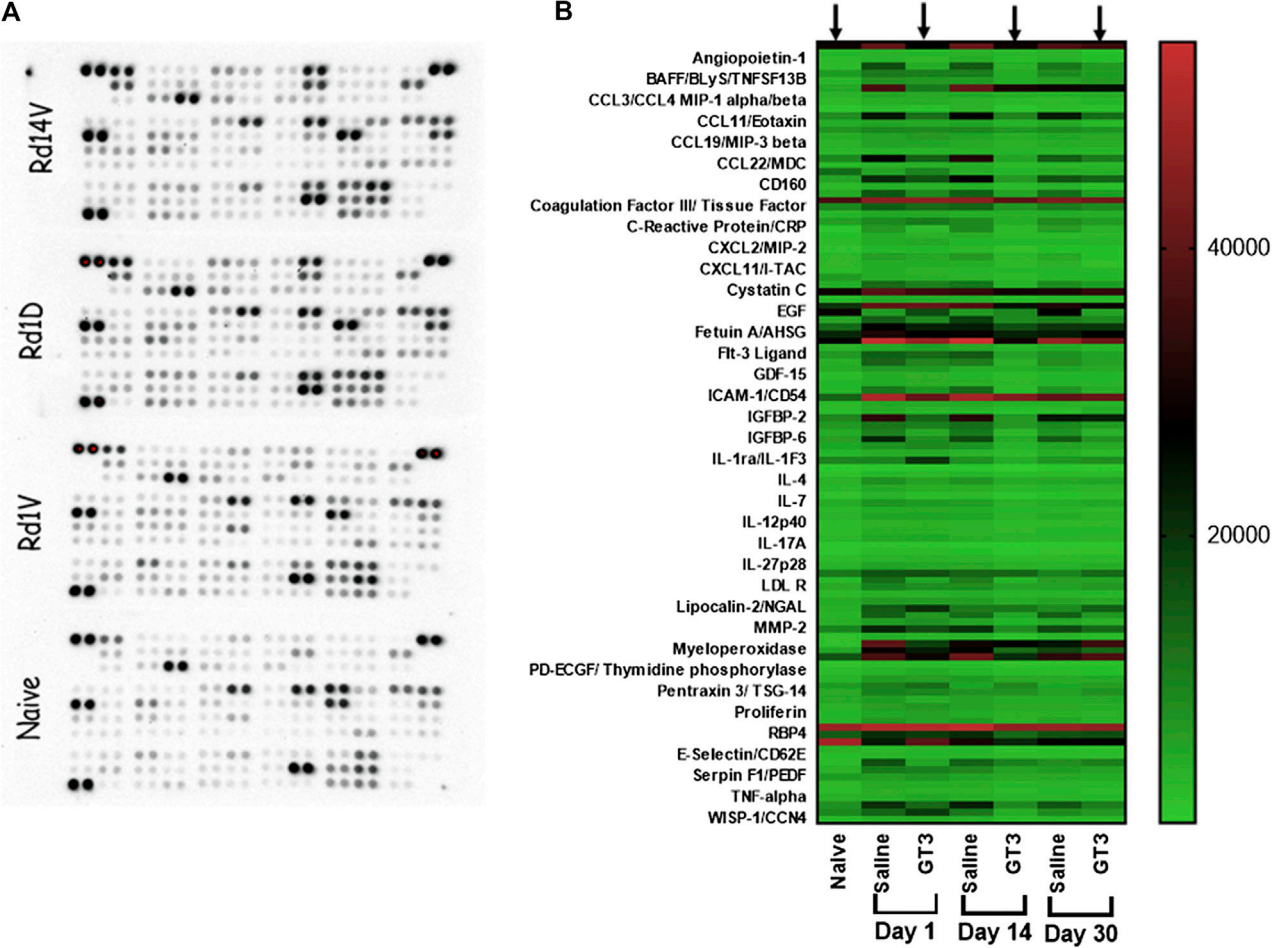
Lung lysates were subjected to a protein Profiler Array of total of 111 cytokines by R&D Systems, a membrane-based sandwich immunoassay. In the kit the capture antibodies were spotted in duplicate on nitrocellulose membranes which bind to specific target proteins in the sample. Captured proteins are detected with biotinylated detection antibodies and visualized using chemiluminescent detection reagents. Signal produced is proportional to the amount of analyte bound. Samples from three groups–naïve, 16 Gy saline and 16 Gy GT3 treated collected on days 1, 14, 30 post-16 Gy lung-PBI were tested. Representative blots are shown in **(A)**. Normalized data from these blots are then represented as a heat-map in **(B)**. Arrows indicate data for GT3 group on different days having similar pattern as the naïve group.

**FIGURE 5:**
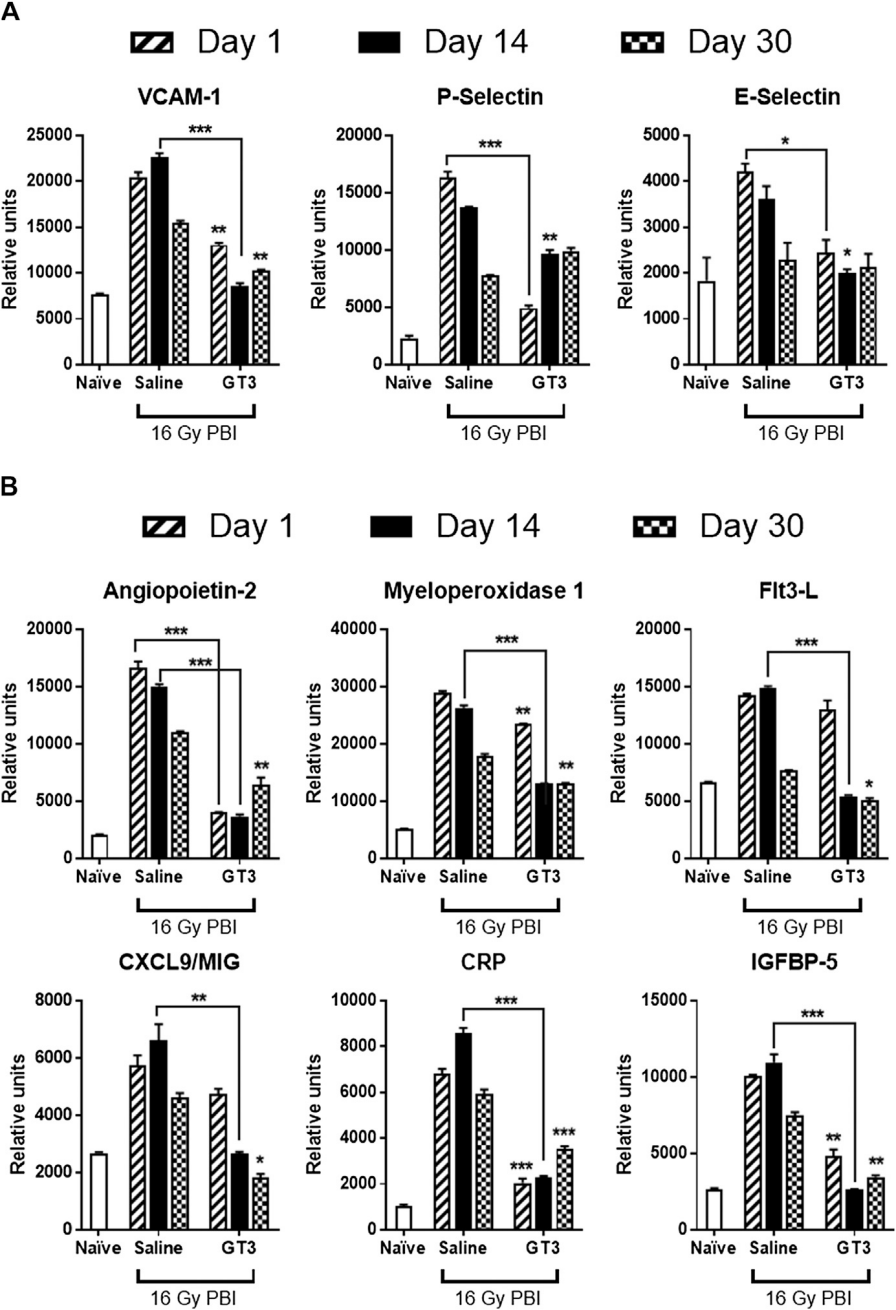
Differential expression of cell adhesion molecules **(A)** and cytokines that were modulation by GT3 **(B)**. **(A)** From the array of 111 cytokines, three cell adhesion molecules (VACM-1, P-selectin and E-selectin) were shortlisted which had shown differential expression in irradiated saline treated group. In the case of VCAM-1 and P-selectin GT3 group showed lower expression closer to the naïve group, whereas saline treated group had much higher protein. **(B)** Six different proteins (Angiopoietin 2, myeloperoxidase 1, Flt3-L, CXCL9, CRP and IGFBP5) were picked based on their higher expression as a result of radiation injury and effective modulation by GT3. Out of the three time-points tested, maximum modulation of hyper-expression was seen on day 14 post-PBI. *p* values: * ≥0.01–0.05, **0.001–0.01, ***≤ 0.01–0.0001.

Lung lysates from all three groups (naïve, 16 Gy PBI saline and GT3) from all three days (days 1, 14, and 30 post-PBI) were subjected to a proteome profiler cytokine array to estimate the levels of cytokines in these samples (Figure 4A). To identify the proteins that were differentially expressed as a result of radiation injury, naïve group was compared to saline treated irradiated group. Effect of pre-PBI treatment by GT3 was evaluated by comparing the expression levels in irradiated saline and GT3 groups. Several cytokines were differentially expressed as a result of radiation injury and inflammation (Figure 4B) in saline treated groups on all days (1, 14, and 30 days post-PBI). Even though expression of some proteins were modulated by GT3 on day 1, many of them stayed similar to that in the control (saline) group, thus showing not much protection. By day 14, the expression pattern in GT3 group looked similar to that in naïve group (Figure 4B arrows). Vascular cell adhesion molecule-1 (VCAM-1), P-selectin, a cell adhesion molecule and E-selectin, the transmembrane receptor on the surfaces of activated endothelial cells had elevated expression after radiation and stayed high until day 30 (the last data point) (Figure 5A) in saline groups. Expression of the cell adhesion molecules is known to rise as response to inflammation (Nakao et al., 1995; Ramsay et al., 1996). VCAM-1 was significantly modulated by GT3 on day 14 (*p* value 0.0005) compared to saline group. On other days as well, the levels of VCAM-1 were significantly lower (*p* value 0.004–0.01) in GT3 groups compared to saline groups. On all three days, levels of P-selectin were significantly lower (*p* values 0.0001–0.01) whereas in the case of E-selectin except for day one (*p* value 0.05) there was not much difference in the two irradiated groups.

Among several other proteins that were differentially expressed, a few (Figure 5B) were short-listed based on the protective effects of GT3. Angiopoietin-2 (Ang-2) levels increase on radiation as seen in the saline treated groups on all three days but GT3 treated animals show significantly lower levels (*p* values 0.0004–0.008) of this protein in their lung. Similar results (*p* value ≤ 0.0001) were seen in the case of C - reactive protein (CRP), the inflammation response marker. Expression levels of Insulin-like growth factor-binding protein 5 (IGFBP-5) were also observed to be elevated on 16 Gy radiation and with GT3 treatment, the levels were kept significantly lower (*p* values 0.0005–0.002), close to the basal level. In the case of Myeloperoxidase 1 (MPO) and chemokine (C-X-C motif) ligand 9 (CXCL9), also known as monokine induced by gamma interferon (MIG), though the expression increased on irradiation, the differential effect between saline and GT3 treated groups was observed only on later time-points on days 14 and 30. Most significant effect on FMS-like tyrosine kinase 3 ligand (Flt3L) was seen on day 14 post-irradiation (*p* value 0.0005).

### Elevated Expression of Phosphorylated Proteins From AKT Signaling Pathway, Amelioration by GT3 Pre-treatment

**FIGURE 6:**
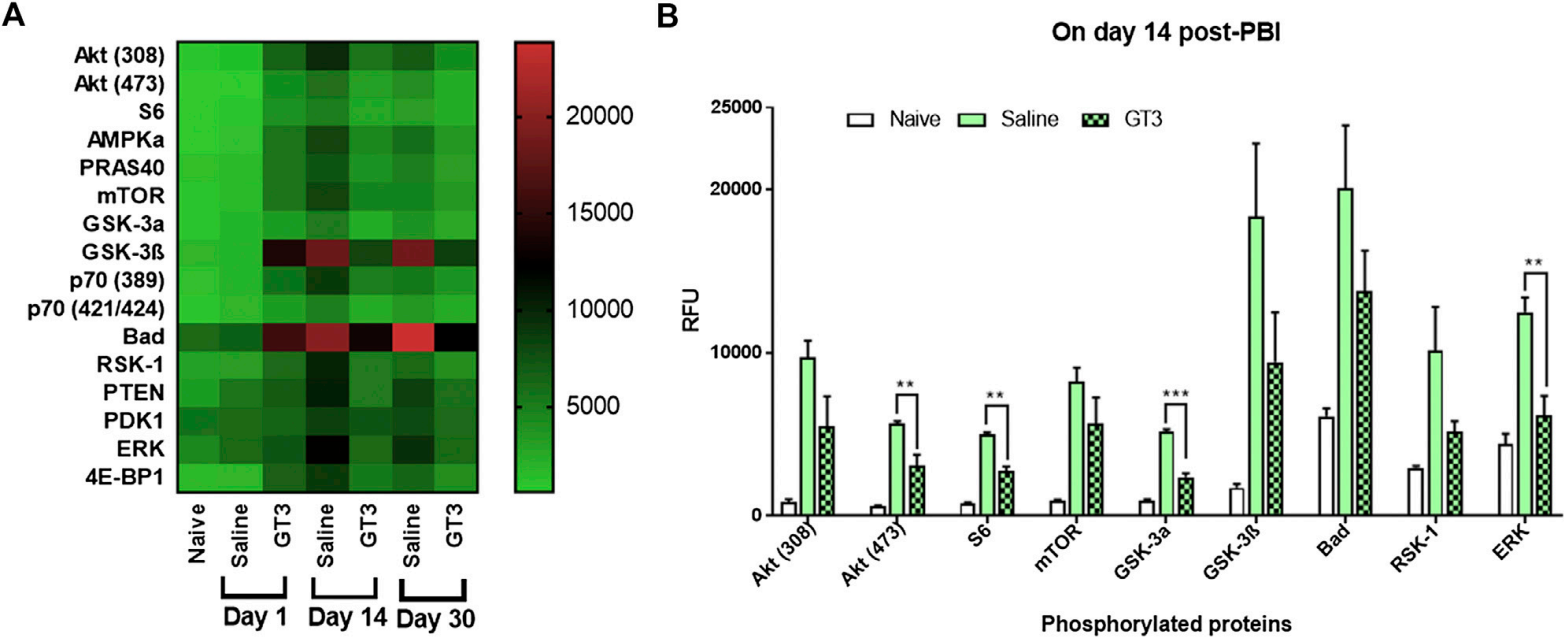
PathScan: Sandwich assay to study differential expression of phosphorylated proteins from AKT pathway. **(A)** A heat map representing the differential expression of different targets screened for naïve and irradiated groups on three time-points. When expression in naïve were compared with the irradiated groups, changes were observed for some of the targets. **(B)** Abrogation of the changes occurring due to radiation injury was seen for some targets in GT3 treated group when compared to saline treated group. Day 14 data is represented as bar graph. **: *p* = 0.001–0.006, ***: *p* < 0.001.

Lung lysates from days 1, 14, and 30 post-PBI prepared as described earlier were subjected to an analysis using an array of phosphorylated proteins from AKT signaling pathway. Expression of these phosphorylated proteins in samples from irradiated groups (saline and GT3) were compared between naïve samples. Sixteen proteins in their phosphorylated forms were assayed for their levels of expression. The changes were not significant on day 1, but on day 14 the saline group looked different from the naïve (Figure 6A). The pattern of expression in the GT3 group was similar to that of the naïve group. By day 30, expression of most proteins in their phosphorylated forms seemed to have recovered from the insult. Out of 16 proteins, 8 of them showed significant increase in phosphorylation with irradiation in the saline group when compared to naïve and phosphorylation of AKT (473), S6, GSK-3a and ERK were significantly inhibited in GT3 group when compared to saline group indicating amelioration by GT3 (Figure 6B).

### Differential Expression or Phosphorylation of Proteins by Immunohistochemistry of Lung Tissue

**FIGURE 7:**
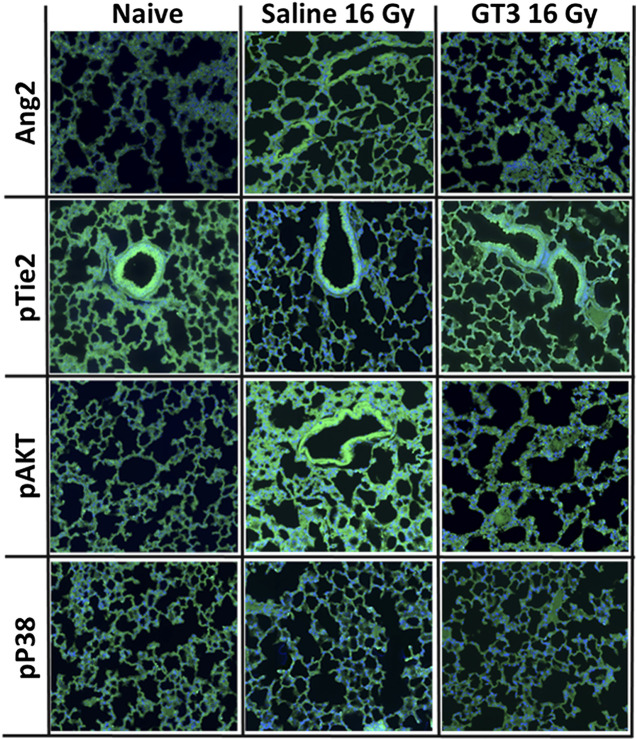
Immunofluorescence (IF) micrographs showing the differential expression of selected proteins. Lung sections from three groups (naïve, day 14 post-PBI 16 Gy irradiated saline and GT3 treated groups) were stained with antibodies against Ang2, pTie2, pAKT and pP38 (green) and DAPI (blue).

Increased Ang-2 and pAKT expression post-PBI in lung by Immunofluorescence were inhibited by GT3 pre-treatment (Figure 7). In the case of Ang-2 there was barely any immunofluorescence in the naïve samples whereas Ang-2 expression increased in the saline treated group however the group administered with GT3 24 h prior to PBI had much lower immunofluorescence. This result corroborated the findings in the cytokine Protein Profiler. As Ang-2 along with Tie-2 is involved in angiogenesis (Mammoto et al., 2012), levels of Tie-2 (data not shown) and phosphorylated Tie-2 (pTie-2) were tested. There was no difference in levels of Tie-2 (total protein) between the three groups (data not shown). The expression for pTie-2 protein was found to be higher in the naïve group as compared to the irradiated saline treated group however in GT3 treated group pTie-2 florescence was higher than in saline group indicating protection against radiation. The phosphorylation of the downstream protein AKT was also increased on PBI in saline treated group compared to naïve and GT3 treated group. We did not see any difference for the phosphorylated P38 among three different groups.

## Discussion

This is a first study that shows the protective role of GT3 against radiation-induced lung injury in mice. Our previous studies have shown that GT3 protected CD2F1 male mice from radiation-induced mortality, improved hematopoietic and gastrointestinal recovery and also induced proliferative cytokines that may play key role in its beneficial effects on the hematopoietic system (Berbee et al., 2009; Ghosh et al., 2009; Kulkarni et al., 2010; Kulkarni et al., 2013). However these studies did not address whether GT3 is also effective in protecting against delayed effects on lungs. The anti-oxidant and anti-inflammatory properties of tocotrienols, specifically GT3 are well documented under various pathological conditions as cancer, heart diseases and diabetes (Aggarwal et al., 2010). Cardioprotective property was proposed to be through cholesterol biosynthesis whereas neuroprotective effects through glutamate-induced activation of c-Src kinase. Attenuation of diabetic conditions have been proposed via multiple pathways some of which are modulation of oxidative-nitrosative stress, suppression of the NF-κB signaling pathway and peroxisome proliferator-activated receptor (PPAR) modulation. Because GT3 is more potent antioxidant than vitamin E alpha tocopherol, we hypothesized that GT3 will be effective in protecting against lung injury.

In a scenario of a nuclear explosion either as an accident or a deliberate attack, the most likely type of exposure to occur is partial body irradiation. There might be some shielding due to the building structures or proximity from the site of explosion (DiCarlo et al., 2011). Studying partial body irradiation has many benefits, one could study detrimental effects on a particular organ system by limiting exposure only to the organ of interest, or as stated in the FDA animal rule, just by attenuating effects of H-ARS, one could study the damage on rest of the body system (FDA, 2015). In this study, we demonstrated significant survival efficacy of GT3 pre-treatment in C3H/HeN male mice when exposed to whole body gamma radiation. In addition, we have shown that pre-treatment of GT3 could protect animals from radiation-induced thoracic injury when animals were exposed to targeted lung radiation in SARRP. C3H/HeN male mice were used in this study as they have been reported to develop early inflammation, alveolitis and fibrosis following lung radiation (O'Brien et al., 2005; Ghita et al., 2019). PBI to thorax was considered to specifically study lung damage without having to deal with either hematopoietic or GI syndrome. The choice of doses were based on the literature where lung damage was seen (O'Brien et al., 2005; Pietrofesa et al., 2013; Barshishat-Kupper et al., 2015). GT3 was administered 24 h prior to radiation exposure based on previously shown optimal time of administration (Ghosh et al., 2009). There were no surviving animals post-TBI when GT3 was administered 48, 8, 4, and 2 h prior to radiation.

We showed that radiation exposure to the thorax doesn’t affect the peripheral blood parameters as expected. Even femoral bone marrow cells did not seem to have been affected as they were spared from radiation exposure. On the contrary, where the sternum was exposed to radiation, cellularity of the sternal bone marrow was severely depleted in vehicle treated animals. Sixteen Gy is a supralethal dose for C3H/HeN mice with respect to TBI and is expected to be severely damaging the bone marrow cells. But GT3 pre-treatment seems to have assisted accelerated recovery from this damage by day 30 post-PBI. In addition to bone marrow cells, the other rapidly dividing cells would be crypts in the jejunum. As the abdominal area was spared from radiation, jejunal crypts were not damaged in radiated groups when compared to naïve animals. Though heart was exposed to radiation, neither epicardial thickening, myocardial fibrosis, nor coronary artery disease was detected in the histopathological evaluation in the duration of the study (30 days). It is possible that injuries to the heart were not evident in this early time point but would have been observed as a delayed effect. In lungs, infiltration of neutrophils, macrophages, lymphocytes causing congestion after radiation (both 14 and 16 Gy), resulted in the thickening of alveolar septa which in turn lead to edema in radiated vehicle treated animals. With GT3 pre-treatment, in the early time-points, an increase in pro-inflammatory cytokines and hematopoietic growth factors such as G-CSF has been shown in irradiated as well as non-irradiated mice which in turn results in increase in neutrophils (Kulkarni et al., 2012). This could be one of the possible reason for a higher inflammatory score in 16 Gy GT3 group compared to saline group on day 1. The protective properties of GT3 were seen at all three time points days 1, 14, and 30 post-PBI, but the greatest difference was observed on day 14.

Role of Vascular cell adhesion protein 1 (VCAM-1) in inflammation and lung injury is well documented (Epperly et al., 2002; Agassandian et al., 2015). As early as day 1 post-PBI the expression levels of VCAM-1 were seen to be elevated in saline group compared to naïve. Though elevated levels of VCAM-1 have been associated with pulmonary fibrosis (Agassandian et al., 2015) its hyper-expression was seen in the early time points (days 1 and 14 post-PBI). The other two cell adhesion molecules E-selectin and P-selectin which are known to be elevated as a result of inflammation and radiation injury (Nakao et al., 1995; Ramsay et al., 1996), were found to be elevated in the saline group. GT3 could abrogate the levels of P-selectin much more effectively as compared to E-selectin. In all these three biomarkers day 14 seemed to be an important time point as the effectiveness of the countermeasure was seen maximum on this day.

Among the other biomarkers tested, Ang-2 expression was found to be significantly increased in saline group following radiation. Pre-treatment with GT3 was found to ameliorate the effect of radiation injury from early (day 1) to later times (day 30). Several other proteins showed significantly higher expression in lung tissue following radiation. Flt-3 ligand is a known biomarker of hematopoietic injury (Kumar et al., 2018), expression of which was abrogated by GT3. Inflammatory biomarker C-reactive protein (CRP) has also been shown to be an effective indicator of possible radiation pneumonitis in humans (Bai et al., 2019) which was also observed as more than 6 fold increase of the protein in saline treated animals. This increased expression is immediate as of day 1 post-PBI and stayed high even on day 30. On the other hand GT3 kept the levels significantly lower. Upregulation of pro-inflammatory chemokines induced by gamma interferon (MIG/CXCL9) has been shown in various organs as a result of radiation injury (Malik et al., 2010; Lierova et al., 2018). Over expression of Insulin like Growth Factor Binding Protein 5 (IGFBP-5) has been correlated with cellular senescence (Kim et al., 2007) and also identified as profibrotic factor which could be an important target for antifibrotic therapies in the case of Idiopathic Pulmonary Fibrosis (Sureshbabu et al., 2011). Exposure to 16 Gy radiation resulted in increased expression of IGFBP-5 in lung tissue of animals treated with saline. On the other hand, GT3 treated group showed similar levels of the protein as in the naïve, healthy animals. Myeloperoxidase (MPO) has a significant role in inflammatory diseases (Malle et al., 2007; Nussbaum et al., 2013). Radiation insult to the lungs resulted in neutrophil accumulation indicated by the combined alveolitis score, which in turn resulted in significant overexpression of MPO saline treated group. GT3 was able to abrogate the effect on days 14 and 30 post-PBI. Some of the proteins discussed above (Ang-2, MIG, and IGFBP-5) would lead to changes in the AKT inflammatory pathway. Activation of phosphorylation of AKT as a result of radiation exposure has been previously studied in endothelial cells (Edwards et al., 2002; Zingg et al., 2004). Systematic characterization using specific inhibitors led to the role of various growth factors and protein kinases triggering the signal transduction resulting in the increase in the down-stream AKT phosphorylation (Edwards et al., 2002; Zingg et al., 2004). In this study, the phosphorylation of the AKT pathway proteins was studied with respect to differential effects due to radiation injury as well as abrogation of this effect due to GT3 pre-treatment. Delta tocotrienol, another vitamin-E isomer, has been shown to have a radioprotective effect through stimulation of ERK activation-associated mTOR survival pathways (Li et al., 2010).

Authors have shown that proteins that regulate angiogenesis, Ang-1 and Ang-2 have opposing functions in inflammation where Ang-1 mitigates vascular inflammation and leakage, and Ang-2 sensitizes the endothelium to inflammatory cytokines (Makinde and Agrawal, 2011; Gavalas et al., 2013). Downstream of Tie-2 protein is known to regulate inflammation by inhibition of surface adhesion molecule expression ICAM-1 and vascular cell adhesion molecule-1 VCAM-1 (Kim et al., 2001). Increased expression of these cell adhesion molecules was observed in saline treated mice following lung radiation and was inhibited to some extent by GT3 treatment.

**FIGURE 8:**
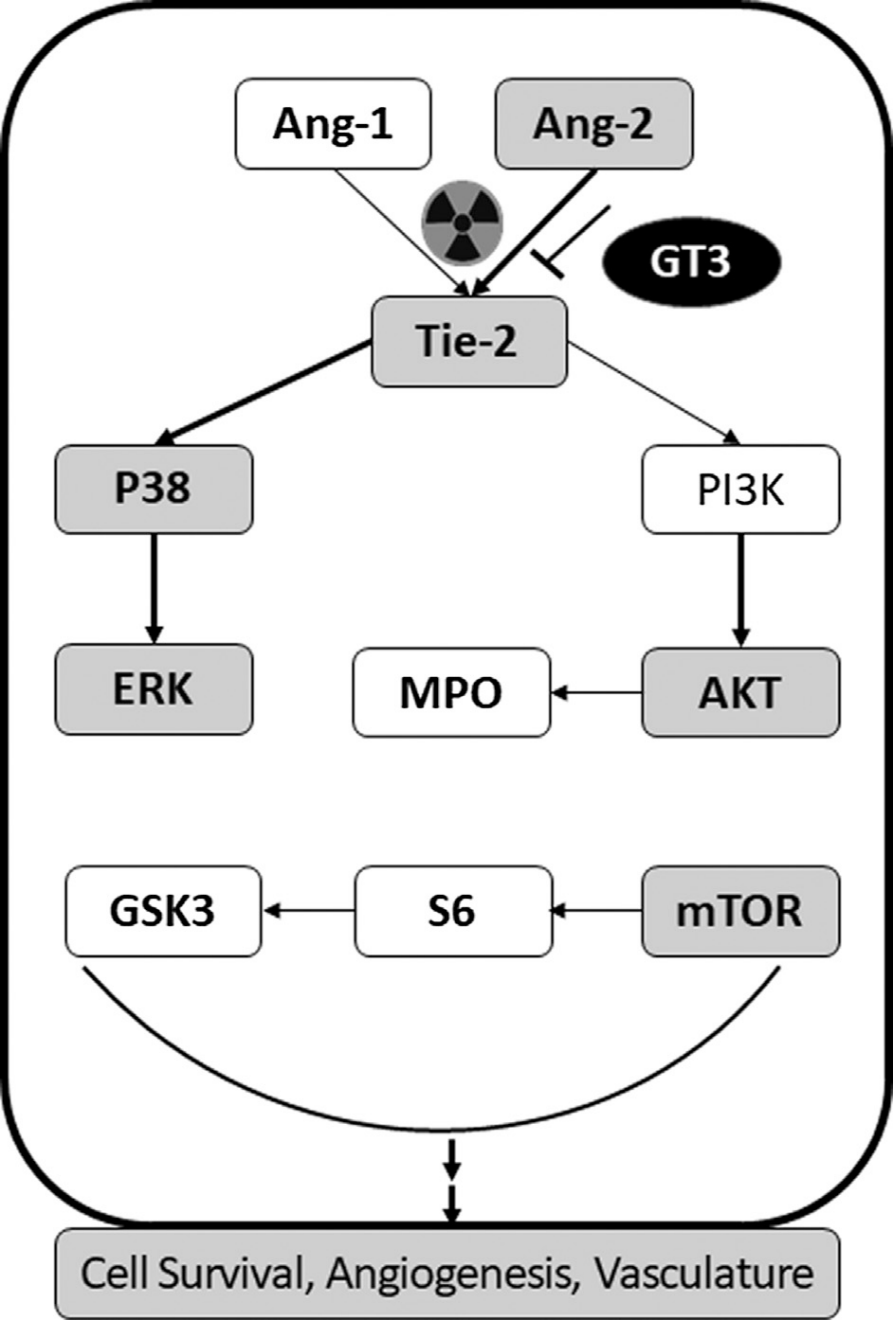
Schematic of the proposed mechanisms of action of GT3. From various assays and methods of detection determining the differential expression of various target proteins from Ang2-Tie2 pathway affecting the downstream pathways AKT and P38 pathways resulting an ultimate effect on cell survival, angiogenesis and vasculature of the lung tissue.

Based on these results as described above, we propose the possible mechanism of action of GT3 in protecting lungs from radiation injury to be via Ang-2/Tie-2 signaling pathway (Figure 8). This was validated by Ang-2 immunohistochemistry (IHC) of lung tissue from naïve and the two irradiated groups (saline and GT3 treated) where it was clear that the phosphorylation of Tie-2 was inhibited as a result of overexpression of Ang-2 which in turn affected the downstream phosphorylation of AKT as observed both by sandwich immune assay and IHC. On the contrary, there was no effect seen in the phosphorylation of P38 in lung tissue. There was no change in the expression levels of Tie-2 protein on radiation (data not shown).

In conclusion, the pre-treatment of GT3 confers protection to lungs by restoring cellularity and megakaryocytes in sternal bone marrow and lowering the occurrence of alveolitis on day 14 post-PBI. As a result of radiation, there is an increase in the expression of Ang-2 which in turn inhibits phosphorylation of Tie-2. This further increased the phosphorylation of AKT cascading the detrimental effects to downstream processes. Prophylactic administration of GT3 to animals before thoracic radiation, regulated Ang-2/Tie-2/ERK/AKT protein expression. Therefore, we propose a possible mechanism of action of GT3 to be via Ang-2-Tie-2 pathway leading to AKT/ERK pathway affecting the cell survival/angiogenesis.

## Data Availability

The raw data supporting the conclusions of this article will be made available by the authors, without undue reservation.
