# Corrigendum: Gamma Tocotrienol Protects Mice From Targeted Thoracic Radiation Injury

**DOI:** 10.3389/fphar.2021.785165

**Published:** 2021-11-29

**Authors:** Vidya P. Kumar, Sasha Stone, Shukla Biswas, Neel Sharma, Sanchita P. Ghosh

**Affiliations:** Armed Forces Radiobiology Research Institute, Uniformed Services University of the Health Sciences, Bethesda, MD, United States

**Keywords:** gamma tocotrienol, partial body irradiation, lung injury, SARRP, small animal radiation research platform, radiation injury to normal lungs, prophylactic treatment, thoracic irradiation

In the original article, we neglected to include the funder “US Department of Defense Threat Reduction Agency grant H.10016_09_AR_R to SG, administered by The Henry M. Jackson Foundation for the Advancement of Military Medicine, Inc.”

In the original article, we missed mentioning that we also used GT3 from American River Nutrition (Hadley, MA, United States). A correction has been made to **Materials and Methods, GT3 Formulation**:

“Pure GT3 was obtained from Yasoo Health Inc. (Johnson City, TN, United States) (Ghosh et al., 2009) and American River Nutrition (Hadley, MA, United States). GT3 and Tween80® (final concentration 5%) were dissolved separately in small volume of ethanol (to enable uniform mixing) and mixed together and then spin-dried under vacuum. Required volume of saline was added to the tube to achieve a final concentration of either 100 or 200 mg GT3 in 0.1 ml.”

Furthermore, we missed acknowledging Dr. Kushal Chakraborty, and missed mentioning the funding agency US Department of Defense Threat Reduction Agency grant H.10016_09_AR_R. A correction has been made to **Acknowledgements**:

“The authors gratefully acknowledge pathologists LTC Culp (USUHS) for histopathological evaluations of slides, the dosimetrists and SARRP operators Sungyop Kim and Dr. David A. Schauer, Kefale Wuddie and Zemenu Aschenake for animal care and manipulations, Dr. Kushal Chakraborty and Betre Legesse for technical assistance, and Anne Trias (American River Nutrition) for providing GT3. The opinions and assertions expressed herein are those of the authors and do not necessarily reflect the official policy or position of the Uniformed Services University or the Department of Defense.”

The authors apologize for this error and state that this does not change the scientific conclusions of the article in any way. The original article has been updated.

