# Supplementary material for: Gamma Tocotrienol Protects Mice From Targeted Thoracic Radiation Injury
Source: Front Pharmacol. 2020 Nov 12;11:587970. doi: 10.3389/fphar.2020.587970 (PMC7748112; doi:10.3389/fphar.2020.587970)
Supplement: Supplementary file 1 [file table1.docx]

**Figure legends**

Fig 1. Survival of C3H/HeN male mice following total-body irradiation at an estimated rate of 0.6 Gy/ min and SC administration of a single dose of either GT3 or saline as vehicle at 24 h prior to TBI. (A) Kaplan-Meier survival curves were plotted using GraphPad software; n=16 mice per group and trend in survival at 8. 5 Gy is compared between vehicle (○) and GT3 (200 mg/ kg) group (●) (log-rank test p < 0.0001). (B) Dose response in terms of radiation doses (7.5, 8, 8.5 and 9 Gy) and doses of GT3 (100 and 200 mg/kg) were evaluated in a 30-day survival study comparing to saline treated group (n=16 / dose of radiation/ treatment group).

Fig 2. GT3 treatment promoted sternal bone marrow hematopoietic cell recovery after 16 Gy PBI (to thorax) when administered 24 h prior to PBI. Representative sternal bone marrow sections are shown for naïve (A), and from saline (C) and GT3 (D) treated 16 Gy irradiated mice from day 30 post-PBI. Bone marrow megakaryocyte numbers were quantitated from histological sections from days 1, 14 and 30 post-PBI (B). Significant increase in bone marrow cellularity and megakaryocytes (B) were observed on day 30 post-PBI in the GT3 treatment group. Data represented are mean ± standard error of the mean (SEM) for n=12 mice.

Fig 3. Histopathological evaluation of lungs irradiated at 14 and 16 Gy. Representative H&E stained lung sections from 16 Gy irradiation groups in (shown as 10X and 40 X magnification) (A) were evaluated for signs of irradiation effects such as characteristic edema of the aveolar septa, type II pneumocyte hyperplasia, monocytic infiltration and fibrosis of alveolar septa, and hyaline membranes that line alveoli; and pulmonary fibrosis. (B) Thickening of alveolar septa (increased numbers of neutrophils, macrophages, lymphocytes, congestion, and edema) was scored as: WNL = 0, 1-33% =1, 34-66% =2, 67-100% = 3. Hemorrhage was grade ‘0’ if not present and ‘1’ if present regardless of amount.
Severe damage to the lung tissue on irradiation was observed on all three days and in both 16 Gy and 14 Gy irradiation doses in saline treated groups. Lung tissue from GT3 treated groups showed less damage and recovery by day 30.

Fig 4. Lung lysates were subjected to a protein Profiler Array of total of 111 cytokines by R&D Systems, a membrane-based sandwich immunoassay. In the kit the capture antibodies were spotted in duplicate on nitrocellulose membranes which bind to specific target proteins in the sample. Captured proteins are detected with biotinylated detection antibodies and visualized using chemiluminescent detection reagents. Signal produced is proportional to the amount of analyte bound. Samples from 3 groups – naïve, 16 Gy saline and 16 Gy GT3 treated collected on days 1, 14, 30 post-16 Gy lung-PBI were tested. Representative blots are shown in (A). Normalized data from these blots are then represented as a heat-map in (B). Arrows indicate data for GT3 group on different days having similar pattern as the naïve group.

Fig 5. Differential expression of cell adhesion molecules (A) and cytokines that were modulation by GT3 (B). (A) From the array of 111 cytokines, three cell adhesion molecules (VACM-1, P-selectin and E-selectin) were shortlisted which had shown differential expression in irradiated saline treated group. In the case of VCAM-1 and P-selectin GT3 group showed lower expression closer to the naïve group, whereas saline treated group had much higher protein. (B) Six different proteins (Angiopoietin 2, myeloperoxidase 1, Flt3-L, CXCL9, CRP and IGFBP5) were picked based on their higher expression as a result of radiation injury and effective modulation by GT3. Out of the three time-points tested, maximum modulation of hyper-expression was seen on day 14 post-PBI. P values: * ≥0.01 – 0.05, **0.001- 0.01, ***≤ 0.01 – 0.0001

Fig 6. PathScan: Sandwich assay to study differential expression of phosphorylated proteins from AKT pathway. (A) A heat map representing the differential expression of different targets screened for naïve and irradiated groups on three time-points. When expression in naïve were compared with the irradiated groups, changes were observed for some of the targets. (B) Abrogation of the changes occurring due to radiation injury was seen for some targets in GT3 treated group when compared to saline treated group. Day 14 data is represented as bar graph. **: p=0.001 – 0.006, ***: p<0.001

Fig 7. Immunofluorescence (IF) micrographs showing the differential expression of selected proteins. Lung sections from three groups (naïve, day 14 post-PBI 16 Gy irradiated saline and GT3 treated groups) were stained with antibodies against Ang2, pTie2, pAKT and pP38 (green) and DAPI (blue).

Fig 8. Schematic of the proposed mechanisms of action of GT3. From various assays and methods of detection determining the differential expression of various target proteins from Ang2-Tie2 pathway affecting the downstream pathways AKT and P38 pathways resulting an ultimate effect on cell survival, angiogenesis and vasculature of the lung tissue.

Suppl Fig 1. Representative images of three mice taken from the Portal imaging camera which provided a fluoroscopic X-ray image using a very short exposure time at 100 kv, 4.0 mA. This 2 D X-ray image assisted in confirming the field of irradiation (lungs) prior to start of actual radiation at either 14 or 16 Gy.

Suppl Fig 2. Effect of PBI on peripheral blood cells (white blood cells (WBC), neutrophils (NEU), platelets (PLT) and RBCs) of irradiated (16 Gy PBI) mice treated with vehicle saline (orange bars) and GT3 (green bars) was compared to the cell numbers in naïve animals (n=12/ group). Either saline or GT3 at 200 mg/kg was administered 24 h prior to irradiation. Day 0 represents day of irradiation. Data represented are mean ± standard error of the mean (SEM) for n=12 mice. There was no significant difference seen in the cell numbers between the irradiated groups and naïve group. As most of the bone marrow except the sternal bone marrow, was spared from irradiation, effect of irradiation was expected to be minimal on the peripheral blood cells.

Suppl Fig 3. Effect of 14 and 16 Gy lung- PBI on femoral bone marrow. During lung-PBI, femurs were not exposed to radiation. Effect on the hematopoietic progenitor cells (Colony forming units) was estimated by assessing the clonogenic potential of bone marrow cells by CFU assay. Colony forming units (CFU) were assayed on days 1 and 30 after exposure. Cells from three femurs were pooled, counted, and each sample plated in duplicate to be scored 14 days after plating. Data are expressed as mean ± Standard error of mean (SEM). No statistically significant difference was determined between irradiated and naïve groups.

Suppl Fig 4. Histopathological evaluation of jejunum (A) and heart (B). (A) H&E stained cross sections of jejunum from naïve and irradiated groups were evaluated for structural damage to the intestinal tissue in terms of number of viable crypts (defined as crypts containing 10 or more adjacent, chromophilic non-Paneth cells). Overall cellularity and proliferating cells in the villi were also compared between the groups. There was no damage observed to the intestinal tissue from the irradiated groups as expected. (B) Heart sections were evaluated for signs of irradiation effects which include epicardial thickening and fibrosis, myocardial fibrosis, and coronary artery disease. The pericardium was not available to evaluate in any of the histologic sections. No significant damage was noticed in the irradiated tissue when compared to naïve. Representative sections are shown in the figure.
