# Supplementary figures and images for: Gamma Tocotrienol Protects Mice From Targeted Thoracic Radiation Injury
Source: Front Pharmacol. 2020 Nov 12;11:587970. doi: 10.3389/fphar.2020.587970 (PMC7748112; doi:10.3389/fphar.2020.587970)

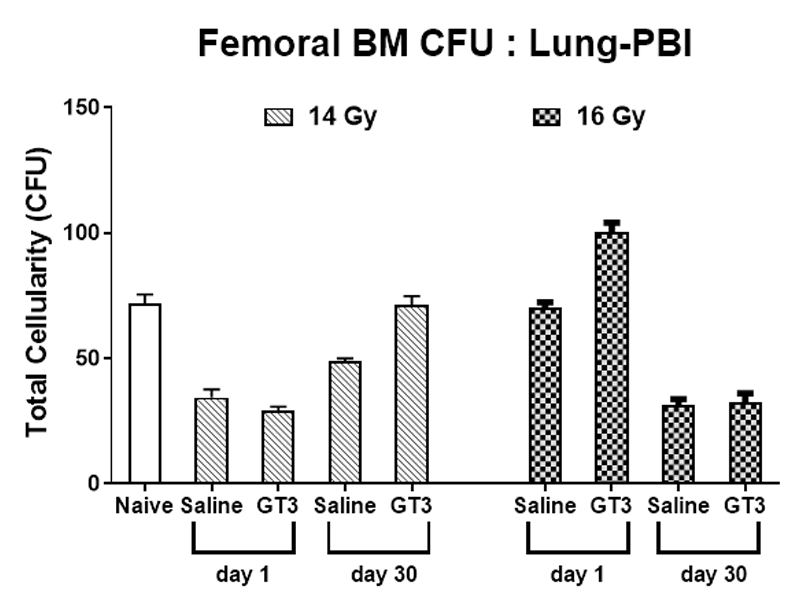

Supplement: Supplementary file 2 [file image3.tif]

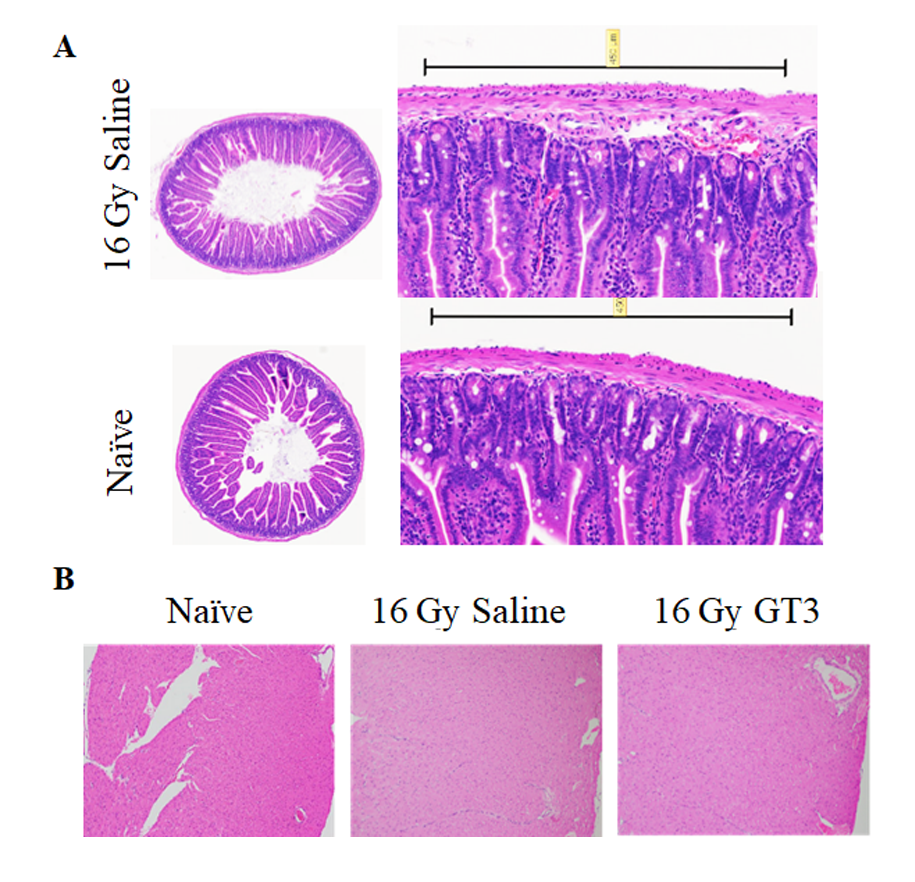

Supplement: Supplementary file 3 [file image4.tif]

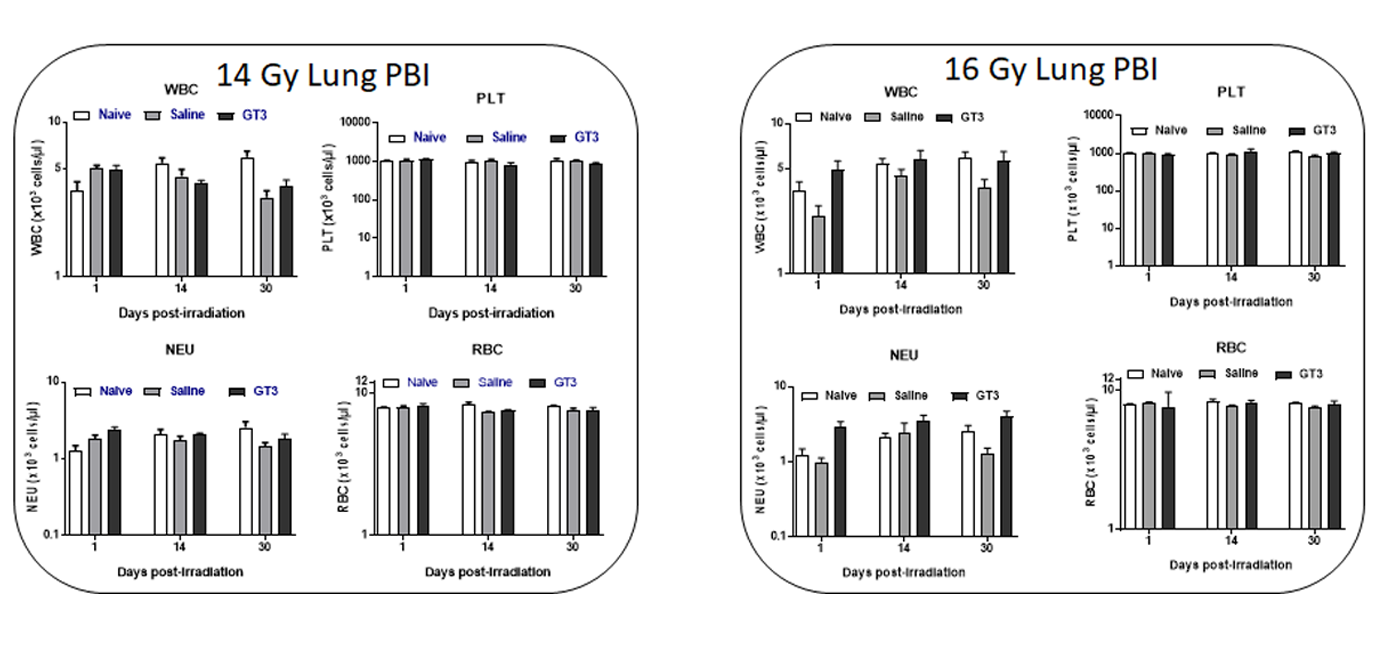

Supplement: Supplementary file 4 [file image2.tif]

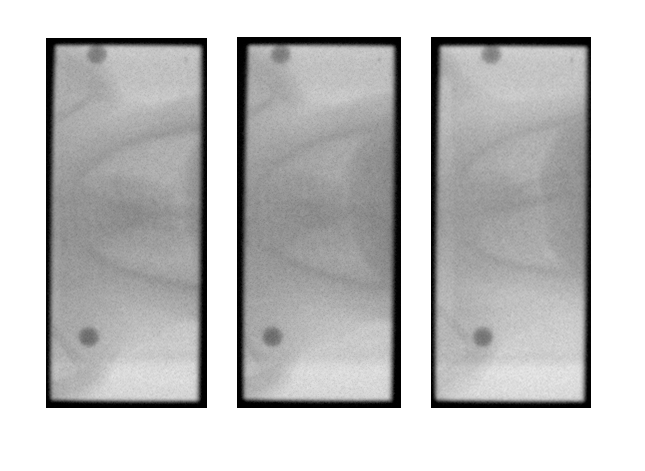

Supplement: Supplementary file 5 [file image1.tif]
